# BinAligner: a heuristic method to align biological networks

**DOI:** 10.1186/1471-2105-14-S14-S8

**Published:** 2013-10-09

**Authors:** Jialiang Yang, Jun Li, Stefan Grünewald, Xiu-Feng Wan

**Affiliations:** 1 Systems Biology Laboratory, Department of Basic Sciences, College of Veterinary Medicine, Mississippi State University, Mississippi State, MS 39762, USA; 2 CAS-MPG Partner Institute for Computational Biology, Key Laboratory of Computational Biology, Shanghai Institutes for Biological Sciences, Chinese Academy of Sciences, Shanghai 200031, China

## Abstract

The advances in high throughput omics technologies have made it possible to characterize molecular interactions within and across various species. Alignments and comparison of molecular networks across species will help detect orthologs and conserved functional modules and provide insights on the evolutionary relationships of the compared species. However, such analyses are not trivial due to the complexity of network and high computational cost. Here we develop a mixture of global and local algorithm, BinAligner, for network alignments. Based on the hypotheses that the similarity between two vertices across networks would be context dependent and that the information from the edges and the structures of subnetworks can be more informative than vertices alone, two scoring schema, 1-neighborhood subnetwork and graphlet, were introduced to derive the scoring matrices between networks, besides the commonly used scoring scheme from vertices. Then the alignment problem is formulated as an assignment problem, which is solved by the combinatorial optimization algorithm, such as the Hungarian method. The proposed algorithm was applied and validated in aligning the protein-protein interaction network of Kaposi's sarcoma associated herpesvirus (KSHV) and that of varicella zoster virus (VZV). Interestingly, we identified several putative functional orthologous proteins with similar functions but very low sequence similarity between the two viruses. For example, KSHV open reading frame 56 (ORF56) and VZV ORF55 are helicase-primase subunits with sequence identity 14.6%, and KSHV ORF75 and VZV ORF44 are tegument proteins with sequence identity 15.3%. These functional pairs can not be identified if one restricts the alignment into orthologous protein pairs. In addition, BinAligner identified a conserved pathway between two viruses, which consists of 7 orthologous protein pairs and these proteins are connected by conserved links. This pathway might be crucial for virus packing and infection.

## Background

In the context of system biology, the concept of network is widely used in representing the interactions between various biological macromolecules. Several distinct types of networks have been modeled at molecular level, such as protein-protein interaction (PPI) networks [1], gene regulatory networks [2], metabolic networks [3], and signal transduction networks [4]. Comparative analyses of these networks can facilitate the identification of conserved components across biological systems and further inference of the biological functions of these components.

A biological network is commonly represented as an undirected graph, in which each vertex corresponds to a biomolecule, e.g. protein, and each edge denotes an interaction between two biomolecules. Conceptually, *network alignment *is to compare and align the vertices of two or more networks to identify subnetwork(s) with similar vertices, which could share alike functions, resembling structure, or common evolutionary history. In recent years, with the development of high-throughput experimental techniques such as the yeast two-hybrid system [5] and co-immunoprecipitation [6], the amount of biological networks has been increasing rapidly, leading to a huge demand for efficient network alignment methods and tools. Because network alignment is in principle an NP-complete problem [7], devising reliable and fast network alignment heuristics has become one of the foremost challenges for network alignments.

A number of network alignment methods have been developed in the past decade [8-16]. Similar to sequence alignment, network alignment methods can be characterized as either global network alignment or local network alignment. *Global network alignment *is to force the alignment to span the entire set of vertices, which can provide insightful views of similarities and differences cross species at the systemic level and help identify functional orthologs. In contrast, *local network alignment *only identifies highly similar subnetworks, which are more likely to be functional components such as pathways.

A pioneering work for global network alignment is IsoRank [14], which adopts a philosophy similar to Google PageRank, that is, a match between two vertices is good if the neighbors of these two vertices matched well. Based on this hypothesis, the global network alignment problem is transformed into an eigenvector problem. A more recent algorithm GRAAL [15] represents the structural information of any vertex by a vector, which records the potential hits of special structures called graphlets in its neighborhoods. By comparing the pairwise similarity between the representing vectors, a global pure graph structure alignment is achieved. Alternatively, the global network alignment problem is transferred into a linear or quadratic integer programming problem, and solved by linear relaxation [17], Lagrangian relaxation [16] or ILOG CPLEX [18]. However, these methods either restrict the alignment into orthologous candidates by setting the score of non-orthologous pairs to be *−∞ *or focus too much on graph structural information. As a consequence, the resulting alignments need to be further optimized.

On the other hand, previous work on PPI networks has been mostly focused on local alignments. PathBLAST [8,9] incorporates the idea of BLAST E-value with PPI network information to identify highly conserved pathways and complexes. By taking into account the duplication/divergence evolutionary model of protein-protein interactions, MaWISH [10] transforms the local network alignment problem into a maximum weight induced subgraph problem and solves the problem in a greedy manner. Graemlin [11] identifies conserved dense subnetworks by comparing the probabilities that a module is under evolutionary constraints and under no evolutionary constraints. Similarly, by comparing the network evolutionary model with random model, Graph Alignment [12,13] presents a complex scoring system on orthologous pairs, non-orthologous pairs, edge matches and mismatches, based on which a local alignment algorithm is designed. These local alignment methods can lead to local optimality because they are generally restricted to subnetworks (e.g. pathways and cliques).

To overcome the limitations of current network alignment algorithms, here we propose a new mixture network alignment method for BIological Network ALIGNment, so called BinAligner. To integrate both local and global network alignments, BinAligner constructs a pairwise similarity matrix between two networks based on three types of similarity scores derived from vertices (e.g. single node comparison based on sequence information), 1-neighbor alignment (e.g. the similarity of two nodes based on the information of their first neighbor subnetworks), and graphlets (e.g. the similarity of *n*-neighborhood subnetworks, *n ≥ *2), which integrate information from both nodes and edges. The introduction of neighborhood subnetworks was based on the hypothesis that the similarity between two vertices across networks would be context-dependent. Then the alignment problem is formulated as an assignment problem, which is solved by the combinatorial optimization algorithm, such as Hungarian method, in polynomial time. The proposed algorithm was applied and validated in aligning the PPI network of *varicella zoster virus *(VZV) and that of *Kaposi's sarcoma associated herpesvirus *(KSHV) [13]. BinAligner outperformed GRAAL [15], Graph Alignment [12,13], and IsoRank [14]. By further checking the biological functions of the aligned pairs, we identified several putative functional orthologous proteins and a conserved pathway between two viruses, which consists of seven orthologous proteins connected by conserved links. This pathway might be crucial for viral packing and infection.

## Methods

Here we use PPI network to illustrate our algorithm. However, this algorithm can be applied to any types of biological networks.

### Mathematical formulation of network alignment

A PPI network is denoted by an undirected graph *G *= (*V*, *E*), where each node *v *∈ *V *represents a protein, and an edge *uv *∈ *E *if there is an interaction between protein *u *and *v*. Given two PPI networks *G *= (*V*, *E*) and *H *= (*U*, *F*), a *network alignment *is defined to be a one-to-one mapping *π *between vertex set *V *and *U*,

(1) π:{i∈V}↦{j∈U}.

The unmapped vertices are assumed to be aligned to virtual dummy vertices. Alternatively, for any *i *∈ *V *and *j *∈ *U*,

(2) $$\pi_{\text{ij}}=\left\{\begin{array}{c} 1,\;\text{if}\;j=\pi \left(i\right),\\ 0,\;\text{otherwise}. \end{array}\right)$$

So a network alignment is achieved if we specify the values of *π_ij _*for all *i *∈ *V *and *j *∈ *U*.

Usually, each network alignment *π *is associated with an alignment score *S *consisting of node score *S_V _*and edge score *S_E_*, which reflect how good vertices and edges being aligned between two networks respectively. In specific, for a pair of vertices *i *and *j*, let *α_ij _*be their sequence similarity score, then $S_{V}=\underset{i\in V}{\sum }\underset{j\in V}{\sum }\alpha_{ij}\pi_{ij}.$ Similarly, let *β_ijkl _*denote the edge score for any 4 vertices *i*, *j*, *k*, *l *such that *i*, *k *∈ *V *and *j*, *l *∈ *U*, then $S_{E}=\sum_{i,k\in V}\sum_{j,l\in U}\beta_{ijkl}\pi_{ij}\pi_{kl}$. And the overall alignment score is

(3) $$S=\underset{i\in V}{\sum }\underset{j\in U}{\sum }\alpha_{ij}\pi_{ij}+\underset{i,k\in V}{\sum }\underset{j,l\in U}{\sum }\beta_{ijkl}\pi_{ij}\pi_{kl}.$$

The objective of global network alignment problem is to find a map *π *to maximize *S *subject to the following constraints,

(4) $$\left\{ \begin{array}{ll} \sum_{i\in V}\pi_{ij}=0\text{ or }1, & \forall j\in U,\\ \sum_{i\in U}\pi_{ij}=0\text{ or }1, & \forall i\in V,\\ \pi_{ij}=0\text{ or }1, & \forall i\in V\text{ and }j\in U. \end{array}\right.$$

The restrictions are obtained since each protein *i *∈ *V *and *j *∈ *U *can at most be mapped once in this framework.

A standard way to improve the speed is to linearize the quadratic objective function by introducing binary decision variables *δ_ijkl _*= *π_ij_π_kl_*. Thus, an equivalent linear integer programming problem is:

(5) $$\underset{\pi }{\text{max}}\underset{i\in V}{\sum }\underset{j\in U}{\sum }\alpha_{ij}\pi_{ij}+\underset{i,k\in V}{\sum }\underset{j,l\in U}{\sum }\beta_{ijkl}{\delta_{i}}_{jkl}$$

subject to

(6) $$\left\{\begin{array}{cc} \underset{i\in V}{\sum }\;\pi_{ij}=0\;\text{or}\;1, & \forall \;j\in U,\\ \underset{i\in U}{\sum }\;\pi_{ij}=0\;\text{or}\;1, & \forall \;i\in V,\\ \delta_{ijkl}\leq \pi_{ij}, & \forall \;i,k\in V\;\text{and}\;j,\;l\in U,\\ \delta_{ijkl}\leq \pi_{kl}, & \forall \;i,k\in V\;\text{and}\;j,\;l\in U,\\ \delta_{ijkl}\geq \pi_{ij}+\pi_{kl}-1, & \forall \;i,k\in V\;\text{and}\;j,\;l\in U,\\ \pi_{ij}=0\;\text{or}\;1, & \forall \;i\in V\;\text{and}\;j\in U\\ \delta_{ijkl}=0\;\text{or}\;1, & \forall \;i,k\in V\;\text{and}\;j,\;l\in U. \end{array}\right)$$

An appropriate scoring scheme is one of the keys to a robust and effective network alignment algorithm. There are several scoring schemes in literatures. For instance, Graph Alignment [12,13] rewards orthologous protein pairs and edge matches, and punishes non-orthologous pairs and edge mismatches by scores based on the log-ratio of the probabilities that they are resulted from evolution or just by chance. Given two pairs of aligned vertices under an alignment *π*, say *j *= *π*(*i*) and *l *= *π*(*k*) with *i*, *k *∈ *V *and *j*, *l *∈ *U*, we say an *edge match *happens if *ik *∈ *E *and *jl *∈ *F *; and an *edge mismatch *happens if *ik *∈ *E *and *jl *∉ *F*, or *ik *∉ *E *and *jl *∈ *F*.

### Construction of similarity matrices

#### Similarity on nodes

We use a matrix *A *to denote the pairwise sequence similarity between vertex set *V *and *U *. In [13], a program called sequenceAlign is developed to calculate the identity score between two proteins and identify orthologous pairs. Let *i *∈ *V *and *j *∈ *U *, we define *A_ij _*= 1 if they are orthologs and *A_ij _*= 0 otherwise.

#### Similarity on 1-neighborhood subnetworks

For networks whose maximum degree is not very large, the linear integer programming method is capable of exactly aligning the 1st neighborhoods of their vertices. The *1st neighborhood *of a vertex *i *is an induced subgraph consisting of all vertices with distance less than or equal to 1 from *i *and the edges between them. Let *i *∈ *V *and *j *∈ *U *be any two vertices in network *G *= (*V*, *E*) and *H *= (*U*, *F *). We use *N_ij _*to denote the best alignment score for the 1st neighborhood of *i *and *j *fixing that *i *is aligned to *j*. *N *is denoted as the similarity matrix on 1-neighborhood subnetworks of *G *and *H*.

Due to the power law nature of PPI networks, there might be a few vertices with large degrees [19]. However, we only need an alignment score, not the exact alignment. Thus, a heuristic method, such as linear or Lagrangian relaxation, is a good alternative in this scenario. In practice, these large-degree vertices make an important role in guiding the alignment. Since the 1st neighborhood alone is too greedy for representing the similarity of two vertices, we incorporate similarities on graphlets to account for higher neighborhoods.

#### Similarity on graphlets (n-neighborhood subnetworks, n ≥ *2*)

**Figure 1:**
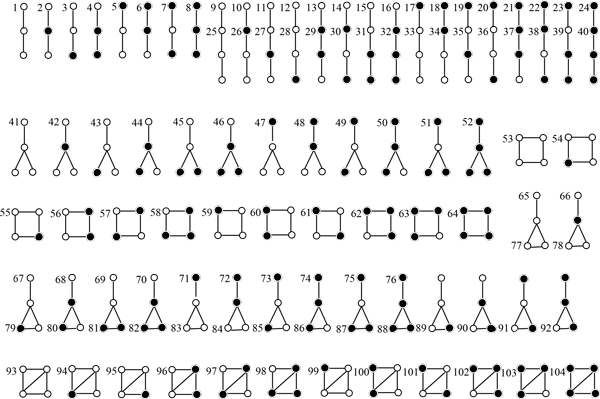
**All 76 graphlets with 104 non-isomorphic positions on 2 to 4 taxa**. A position in a graphlet is denoted by a circle or solid circle: the solid circle position requires the aligned two vertices at this position to be orthologs, whereas the circle position does not have this requirement.

The concept of graphlet and orbit was introduced by Przulj *et al. *[15,20] to measure network local similarities. A *graphlet *is a small connected non-isomorphic subgraph of a large network, in which the non-isomorphic positions are labeled, and a graphlet with an unique labeled position is called an orbit [20]. However, to our best knowledge, the vertex similarity information (e.g. orthology) has not been considered in graphlet definition. In this framework, we explicitly incorporate the vertex similarity information by introducing different types of positions in a graphlet: (1) positions requiring vertex sequence similarity and (2) positions without this requirement. We list in Figure 1 all 76 graphlets containing 104 non-isomorphic orbits on 2 to 4 taxa under symmetry, in which positions requiring vertex sequence similarity are denoted by solid circles, and other positions by normal circles. A weight is also associated to each graphlet to reflect their chances of occurrence. Specifically, let the score of a normal circle be 0, for consistency we define the score of a solid circle and that of an edge between two solid circles to be the scores for orthologous pair and the scores for edge match, respectively, as we defined earlier in the 1st neighborhood alignment. For example, let *α *and *β *be the scores for orthologous pair and edge match, then the first graphlet has weight 2*β *and the 4th graphlet has weight 2(*α *+ *β*). The graphlets and orbits containing only the 1st neighborhood information, complete graph, will be ignored because the information for the 1st neighborhood has already been considered in lst neighbor alignment.

Let  O be a set of orbits. For any o∈O, we say that two networks *G *= (*V*, *E*) and *H *= (*U*, *F *) hit *o *at (*i*, *j*), *i *∈ *V *and *j *∈ *U *, if there is a local alignment A  between *G *and *H *such that

• *i *is aligned to *j*.

• *o *is an induced subgraph of the alignment graph of A  with (*i*, *j*) being placed at the labeled vertex of *o*.

Where the alignment graph of A  is a graph such that: (1) the vertex set consists of all aligned pairs (*k*, *l*) of vertices between *V *and *H*, (*k*, *l*) is dented by a solid circle if *k *and *l *are orthologs and normal circle otherwise; (2) there is an edge between two pairs of aligned vertices (*i*, *j*) and (*k*, *l*) if *ik *and *jl *are connected in *G *and *H *respectively. We use a vector $\overset{\to }{s_{\mathrm{ij}}}$ of dimension 104 to denote the similarity of *i *and *j *on graphlets. Specifically, $\overset{\to }{s_{\mathrm{ij}}}$ [*k*] with 1 *≤ k ≤ *104 counts the number of possible hits of the corresponding graphlet between networks *G *and *H *by fixing that *i *and *j *are located at position *k*. Since some graphlets are contained in other graphlets, only the hits of graphlet with the highest score is counted. For example, if say graphlet 1, 2 and 4 are hit at some pair (*i*, *j*), then only the entry of $\overset{\to }{s_{\mathrm{ij}}}$ at graphlet 4 will be added by one. The graphlet score *B_i_*,*_j _*of a pair (*i*, *j*) is then counted as the weighed sum of the entries in $\overset{\to }{s_{\mathrm{ij}}}$. In general, we use a matrix *B *to denote the similarity of networks *G *and *H *on graphlets.

The three similarity matrices *A*, *N *and *B *are then normalized by the largest entry in them. For simplicity, we still use *A*, *N *and *B *to denote the normalized matrices. Although *A*, *N *and *B *alone already reflects the similarity of each pair of vertices between network *G *and *H*, sometimes better alignment could be retrieved from their weighted combination *C *= *θ*_1 _∗ *A *+ *θ*_2 _∗ *N *+ *θ*_3 _∗ *B *where 0 *≤ θ*_1_, *θ*_2_, *θ*_3 _*≤ *1, *θ*_1 _+ *θ*_2 _+ *θ*_3 _= 1 are the parameters to balance the importance of vertex similarity, 1-neighborhood, and *n *neighborhoods (*n >*= 2).

### Retrieving alignments from similarity matrices

The network alignment *π *from the similarity matrix *C *was generated by solving the following assignment problem:

(7) $$\underset{\pi }{\text{max}}=\underset{i\in V}{\sum }\underset{j\in U}{\sum }C_{ij}\pi_{ij}$$

subject to

(8) $$\left\{\begin{array}{cc} \underset{i\in V}{\sum }\pi_{ij}=1, & \forall j\in U,\\ \underset{i\in U}{\sum }\pi_{ij}=1, & \forall i\in V,\\ \pi_{ij}=0\;or\;1,\;\;\;\;\; & \forall i\in V\;and\;j\in U, \end{array}\right)$$

This assignment problem can be solved by the Hungarian method or ILOG CLPEX in polynomial time. An alternative strategy to retrieve the alignment is to first find high scoring pairs and fix them, then gradually expand the obtained local alignments in their close neighborhoods according to the alignment score defined by *S *in Eqn. 3, until all the vertices are aligned. In this process, some good local alignments and a global alignment are obtained simultaneously.

It is worth noting that both strategies have their advantages and suffer the problem of tie-breaking. To improve the performance, after obtaining the optimal assignment score, we refine the alignment according to the score function *S*. In specific, let ŝ be the optimal assignment score for equation (7). We add a new restriction $\underset{i\in G}{\sum }\underset{j\in H}{\sum }C_{ij}\pi_{ij}=$ ŝ to restrictions (8) and resolve the optimization problem:

(9) $$\underset{\pi }{\text{max}}=\underset{i\in V}{\sum }\underset{j\in U}{\sum }\alpha_{ij}\pi_{ij}+\underset{i,k\in V}{\sum }\underset{j,l\in U}{\sum }\beta_{ijkl}\pi_{ij}\pi_{kl}$$

subject to

(10) $$\left\{\begin{array}{cc} \underset{i\in V}{\sum }\underset{j\in U}{\sum }C_{ij}\pi_{ij}=ŝ, & \\ \underset{i\in V}{\sum }\pi_{ij}=1, & \forall j\in U,\\ \underset{i\in V}{\sum }\pi_{ij}=1, & \forall i\in V,\\ \pi_{ij}=0\;or\;1, & \forall i\in V\;and\;j\in U, \end{array}\right)$$

This process will not increase the running time much because usually the solution space for the assignment problem is small.

### Parameter optimization

A challenging problem is how to specify the parameters *α_ij _*and *β_ijkl_*, that is, the score for node pair (*i*, *j*) and that for the link pair (*ik*, *jl*) for all *i*, *k *∈ *V *and *j*, *l *∈ *U*. Generally, *α_ij _*is positive if protein *i *and *j *are orthologs and negative otherwise. Similarly, *β_ijkl _*is positive if *ik *and *jl *are an edge match and negative if they are an edge mismatch. The values could be evaluated by the probabilities of a node match, mismatch, edge match and mismatch by randomly aligning the two networks. A complicated scoring scheme was shown in GraphAlignment [12], which provides functions to generate reasonable parameters using Bayesian statistics on merely the information of the two networks. We adopt these parameters as follows,

(11) $$\alpha_{ij}=\left\{\begin{array}{ll} 4.4 & i\;\text{and}\;j\;\text{are orthologs},\\ -1.6 & \text{else}, \end{array}\right)$$

and

(12) $$\beta_{ijkl}=\left\{\begin{array}{cc} 1.6 & \text{if}\;ik\;\in \;E\;\text{and}\;jl\in F,\\ -0.3 & \text{else}. \end{array}\right)$$

### Performance assessment of network alignment

For an alignment π:V↦U, two parameters were used to evaluate global network alignment: edge correctness [15] and orthologous percentage. The *edge correctness (EC) *is defined as the proportion of aligned edges in *G *= (*V*, *E*) over the number of edges *|E| *in the network. The *orthologous percentage (OP) *is defined as the number of aligned orthologous pairs over the theoretical maximum number of orthologous pairs being aligned. Both parameters are between 0 and 1, and the larger the better.

We also adopt the geometric random graph model, a widely used theoretical model for PPI networks [15,20-23], to analyze the statistical significance of our edge alignments. In this model, proteins are modeled as existing in a metric space and are connected by an edge if they are within a fixed, specified distance of each other. By this model, let *n*_1 _= *|V | *and *n*_2 _= *|U | *be the number of nodes and *m*_1 _= *|E| *and *m*_2 _= *|F | *be the number of edges of the two networks respectively. The probability *P *of successfully aligning *k *or more edges by chance is

(13) $$P=\overset{m1}{\underset{i=k}{\sum }}\left(\begin{array}{c} m_{2}\\ i \end{array}\right)\left(\begin{array}{c} p-m_{2}\\ m_{1}-i \end{array}\right)/\left(\begin{array}{c} p\\ m_{1} \end{array}\right),$$

where $p=\frac{n_{2}\left(n_{2}-1\right)}{2}$[15]. Usually, *P <*0.025 is considered to be statistically significant, and the smaller is the *P *value, the more significant is the alignment.

In the end, we evaluate the performance of an alignment by exploring functions of aligned proteins. A biologically good alignment should align proteins in one network to those in another with similar functions, and should be able to find some functional orthologs missed by other alignments. In addition, it would be critical if the alignment is capable of finding some common subnetworks between two networks, which might be conserved for some important functions. However, there is no absolute criteria for comparing the protein functions as in most cases the functions of aligned proteins might be not fully known.

## Results

### Benchmark datasets

To validate BinAligner, we perform analysis on aligning PPI networks of two herpes viruses, the VZV, which causes chicken-pox in children, and KSHV, which causes *Kaposi's sarcoma*. These two viruses are both herpes-virus and closely related in evolution. In addition, they are common human pathogens. Although their interactions with human are widely studied, there is relatively little knowledge about protein interactions among these viral proteins. A comparative network study could provide insights on these pathogens.

The interactions of their open reading frames (ORFs) can be found in the supplement of [24]. Similar to Berg and Lässig [13], we construct VZV and KSHV networks by using nodes to denote ORFs and links to denote the interactions between ORFs. The two networks are shown in Figure S1 and S2 (Additional file 1). The graphs are constructed using a free software Graphviz [25].

The VZV network consists of 173 interactions and 76 ORFs, among which 19 ORFs have no interaction and there are 13 self interactions. For convenience, we remove the isolated vertices and self links, and denote the network by a graph *G *= {*V*, *E*}, in which *|V| *= 57 and *|E| *= 160. Similarly, the KSHV network consists of 123 interactions and 84 ORFs, among which 34 ORFs have no interaction and there are 8 self interactions. We denote the network by *H *= {*U*, *F*}, in which *|U| *= 50 and *|F| *= 115. According to the orthologous table in [13], there are 25 orthologous pairs between the ORFs of *V *and *U *if we remove the isolated orthologous ORFs (see Table S1 in Additional file 1). Because several proteins have more than one orthologs, theoretically the maximum number of non-overlapping orthologous pairs in an alignment is 16.

In this study, we developed a new similarity measure so called 1- neighborhood subnetwork, introduced the orthologous information into graphlets (*n*-neighborhood subnetwork, *n ≥ *2), and integrated neighborhood subnetwork and graphlet with conventional sequence similarity. To demonstrate the usefulness of new features and examine the importance of this integrative measure for distance measurement, we compare the alignment results derived from this new measure with those solely based on orthology information or graph structural information. Our results demonstrated that integration of orthologous information, 1-neighborhood subnetwork, and orthologous graphlet scoring scheme, will lead to the best performance in network alignments. Finally, BinAligner was also compared with three widely used network alignment programs, including GRAAL [15], Graph Alignment [12,13], and IsoRank [14].

### Alignments of KSHV and VZV PPI networks solely based on orthologous information

By setting *θ*_2 _and *θ*_3 _to be 0, BinAligner generates an alignment based solely on orthologous information. We list in Table S2 (Additional file 1) the alignment table and also plot the alignment graph with orthologous pairs and matched edges in Figure S3 (Additional file 1) for a better view. This alignment identified 16 orthologous pairs together with 45 matched links, and thus the orthologous percentage is 100% and the edge correctness is 39.1%. Though the largest possible 16 orthologous pairs are aligned, it seems that some of them are misaligned because the alignment is restricted to orthologous pairs and thus the proteins with similar function but low sequence similarity could not be aligned. For example, KSHV ORF67.5 is aligned to VZV ORF49 and KSHV ORF23 is aligned to VZV ORF25. However, by checking the functions, KSHV ORF67.5/VZV ORF 25 are homologs of the HHV-1 protein *UL*33 [13]; VZV ORF49 is likely a myristylated tegument protein [26] and KSHV ORF23 is herpesvirus core gene UL21 family. Obviously, these two pairs are misaligned since ORF67.5 has several sequence orthologs, and sequence information alone cannot distinguish them. As a consequence, some important pathway conserved in KSVH and VZV PPIs are more likely to be broken. Thus, it seems that interaction pattern from link information are necessary to guide orthologous pair alignments when a protein has several orthologous partners (see the results in later section). Another major limitation for orthologous information based alignment is that it is not effective in identifying those functional orthologs with low sequence similarity. In our application, it is not surprising that except for the orthologous pairs, this alignment failed in identifying any other seemingly functional orthologous pairs since the alignment was generated based on only orthologous information.

### Alignments of KSHV and VZV PPI networks solely based on graph structural information

By setting *θ*_1 _and *α_ij _*to be 0, the KSHV and VZV PPI networks were aligned merely using graph structural information. The aligned network has 68 edges (see Figure S4 in Additional file 1). The edge correctness is 59.1%, and P-value is about 6.2 *× *10^−44^. The details for aligned nodes are available in Table S3 (Additional file 1). Surprisingly, no orthologous pair was shown in the alignment network thus the alignment is probably not much biological meaningful. This result suggest that additional biological domain knowledge is crucial to be included to guide a biological network alignment, as different from other non-biological network alignment. Other studies have showed that pure graph structural alignment could be very useful in aligning other types of non-biological networks, such as computer networks and social networks [27].

### Integration of orthologous information and neighborhood subnetwork scoring scheme resulted in the best alignment performance

**Figure 2:**
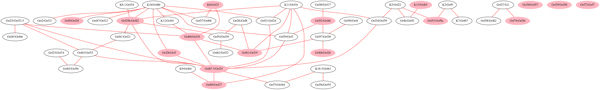
**The alignment graph only containing aligned orthologous pairs and matched edges**. Each node represents a pair of aligned ORFs and the orthologous pairs are shaded.

By combining the orthologous information, 1-neighborhood subnetwork and graphlet, the aligned network between KSHV and VZV PPI networks has the largest possible 16 orthologous pairs and 58 interactions (see Table 1). Thus, the orthologous percentage is 100%, the edge correctness is 50.4%, and the P-value for the edge alignment is about 1.0 *× *10^−32^. The sub alignment graph illustrating the aligned orthologous pairs and matched edges was shown in Figure 2, and the entire aligned graph was available in Figure S5 in Additional file 1).

**Table 1:** The best alignment of KSHV and VZV by BinAligner

KSHV	VZV	orth	KSHV	VZV	orth
ORF2	ORF15	0	ORF58	ORF62	0
ORF6	ORF43	0	ORF59	ORF3	0
ORF9	ORF28	1	ORF60	ORF18	1
ORF23	ORF33.5	0	ORF61	ORF19	1
ORF25	ORF14	0	ORF62	ORF32	0
ORF27	S/L	0	ORF63	ORF33	0
ORF28	ORF1	1	ORF65	ORF56	0
ORF29b	ORF42	1	ORF67.5	ORF25	1
ORF30	ORF57	1	ORF68	ORF26	1
ORF31	ORF24	0	ORF69	ORF27	1
ORF34	ORF59	0	ORF72	ORF7	1
ORF36	ORF8	0	ORF74	ORF36	1
ORF37	ORF68	0	ORF75	ORF44	0
ORF39	ORF50	1	K3	ORF9	0
ORF41	ORF21	0	K5	ORF22	0
ORF45	ORF66	0	K7	ORF67	0
ORF47	ORF12	0	K8	ORF23	1
ORF49	ORF17	0	K9	ORF64	0
ORF50	ORF4	0	K10	ORF60	0
ORF52	ORF46	1	K10.5	ORF61	0
ORF53	ORF9a	1	K11	ORF16	0
ORF54	ORF39	0	K12	ORF41	0
ORF56	ORF55	0	K15	ORF65	1
ORF57	ORF38	0			

The orthologous pairs are marked with 1 in the column orth.

In this aligned network, there is a connected sub alignment graph with 7 pairs of orthologous vertices which is connected by matched links. The pairs (KSHV/VZV) are ORF29b/ORF42, ORF67.5/ORF25, ORF60/ORF18, ORF61/ORF19, K8/ORF23, ORF69/ORF27 and ORF28/ORF1 and their functions are listed in Table 2. As the function all the orthologous pairs are related to virus packing and infection, this pathway might be crucial in both viruses.

**Table 2:** Functions of 7 orthologous protein pairs connecting by matched links

KSHV/ VZV	Function
ORF29b/ORF42 ORF67.5/ORF25 ORF60/ORF18 ORF61/ORF18 K8/ORF23 ORF28/ORF1 ORF69/ORF27	DNA packing proteins DNA packing proteins: UL33-Like Ribonucleotide reductases small subunits, belong to ferritin-like diiron-binding domain Ribonucleotide reductase large units, belong to barrel domain Virus infection Membrane proteins UL31-like proteins but have no known function

In addition, BinAligner also identified some putative functional orthologous pairs with low sequence similarity but with similar function. For example, KSHV ORF56 is aligned to VZV ORF55, their sequence identity is 14.6%, however, functionally they are both helicase-primase subunits. Similarly, KSHV ORF75 is aligned to VZV ORF44, their sequence identity is 15.3%, however they are both tegument proteins. KSHV ORF50 and VZV ORF4 are herpesvirus transcription factors with sequence identity is 11.4%. These putative functional orthologous proteins cannot be identified if we restrict the alignment into orthologous protein pairs as some conventional methods did, which confirms the importance of neighborhood similarity.

### Effectiveness of sequence similarity, 1-neighborhood subnetwork and graphlet

The parameters *θ*_1_, *θ*_2_, and *θ*_3 _balance the contribution of sequence similarity and neighborhood similarities. To test their influences, we compare the number of aligned orthologous pairs and matched edges using different parameters. We test the performances of using (1) only one scheme by setting one parameter to be 1 and the other two to be 0, (2) the combination of two schemes by setting the other parameter to be 0 and (3) the combination of three schemes. The full results are showing in Table S4 (Additional file 1), from which we chose a sub table (see Table 3) to show the importance of the 3 schemes. From the tables, an observation for comparing KSHV and VZV network is that sequence similarity contributes most to the orthologous pairs being aligned, whereas 1-neighbor subnetwork is crucial to the number of matched edges. Graphlets contributes to both, but is not as important as the other two parameters. A possible reason is that we exclude the cliques in the graphlets. The results might be different if we add them back. However, it is beyond this study.

**Table 3:** The influence of balancing parameters on the alignment

*θ*_1_	*θ*_2_	*θ*_3_	nEdge	nOrth
1	0	0	45	16
0	1	0	54	9
0	0	1	53	10
0.9	0.1	0	57	16
0.5	0.5	0	50	16
0.1	0.9	0	56	12
0.9	0	0.1	54	16
0.5	0	0.5	47	16
0.1	0	0.9	50	12
0	0.9	0.1	52	10
0	0.5	0.5	52	10
0	0.1	0.9	54	10
0.9	0.09	0.01	58	16
0.9	0.05	0.05	43	16
0.9	0.01	0.09	51	16

nEdge denotes the number of aligned matched edges; nOrth denotes the number of aligned orthologous pairs.

### Comparison with other algorithms on KSHV and VZV PPI network alignments

In this section, we compared BinAligner with three popular network alignment algorithms IsoRank [14], GRAAL [15] and Graph Alignment [12,13]. Performance evaluation of network alignment is based on the number of orthologous pairs and vertices. The more orthologous pairs, the better performance; the more vertices, the better performance.

For a fair comparison, besides our method, we tune the parameters in IsoRank and GRAAL. IsoRank and GRAAL both have one parameter to balance the node and link contribution. For IsoRank, the parameter is optimized with the range of 0 to 1 with a step of 0.01 and the parameter of GRAAL goes from 0 to 1 with step 0.1. The inconsistency in step size was arisen since the minimum increasing of step is set to be 0.1 in GRAAL. Graph Alignment is parameter-free since it provides some pre-assessing for parameters in its program, however the quality of the alignment seems to be quite dependent on the initial random seed chosen. We run Graph Alignment for 100 times and only record the best alignment. The comparison of all 4 methods are shown in Table 4.

**Table 4:** Comparison of four methods on aligning the PPI networks of KSHV and VZV

Method	nEdge	nOrth	EC	OP	P-value
GRAAL	45	2	39.1%	12.5%	2.6 *× *10^−19^
GraphAlignment	51	9	44.3 %	56.3%	4.3 *× *10^−25^
IsoRank	48	15	41.8%	93.8%	4.1 *× *10^−22^
BinAligner(S)	68	0	59.1%	0	6.2*× *10^−44^
BinAligner	58	16	50.4%	100%	1.0 *× *10^−32^

BinAligner(S) means pure graph structure alignment using BinAligner; nEdge denotes the number of aligned matched edges; nOrth denotes the number of aligned orthologous pairs; EC denotes edge correctness and OP denotes orthologous percentage.

Our results showed that BinAligner achieved the highest number of orthologous protein pairs and matched link pairs. Since GRAAL and GraphAlignment only aligned 2 and 9 pairs of orthologous protein and the aligned interactions are also significantly less than BinAligner, we only compare functionally the alignment by BinAligner (see Table 1) and that by IsoRank (see Table S5 in Additional file 1). The two alignments share almost all orthologous pairs except that BinAligner generates one more orthologous pair KSHV ORFK15/VZV ORF65. ORFK15 is a signal transducing membrane protein and ORF65 is a tegument protein, which immunoprecipitated a 16-kDa protein from the membrane fraction of VZV-infected cells [28]. However, KSHV ORFK15 is aligned to VZV ORF64 by IsoRank where VZV ORF64 is a Gene66(IRS) protein and is by no means to be aligned to a signal transducing membrane protein. In addition, the identified functional orthologous pairs by BinAligner were missed by IsoRank. For example, instead of aligning KSHV ORF56/VZV ORF55, which are both helicase-primase subunits, KSHV ORF56 is aligned to VZV ORF59 by IsoRank, which is an uracil-DNA glycosylase. Since BinAligner also aligned 10 more matched links, so we believe that our alignment is better than that by IsoRank though the two alignments indeed share a lot of aligned protein pairs.

## Discussion

In the study, a pairwise similarity matrix on vertices of two biological networks is constructed from sequence similarity, 1-neighborhood subnetwork, and graphlets with orthologous information. The philosophy is that the similarity of two nodes in different biological networks is reflected successively by their sequence similarity (their own information), similarity of the vertices and edges link to them, and similarity of those indirectly links to them. The closer the vertices and edges are to the compared core vertices, the more impacts they are in reflecting the similarity of the compared vertices. To the best of our knowledge, the 1-neighborhood subnetwork and graphlets with orthologous information have not been studied in the literatures. And our example illustrate that the two similarity measures, especially the 1-neighborhood subnetwork contribute significantly in identifying a good network alignment. In addition, we remove the orthologous information and conducting network structure based alignment, which also show the importance of 1-neighborhood subnetwork similarity in guiding a good alignment. The graphlets with orthologous information are incorporated to account for the information of farther neighborhoods. In this study, 104 graphlets were applied to consider information from up to 3-neighborhood. The global similarity of two proteins is mostly decided by its sequence similarity and then the proteins and interactions close to them. However, the far proteins and interactions may still have indirect influence on them. So it could be beneficial to consider this indirect information. In practice, the best alignment was achieved by combining the three similarity measures.

Similar to sequence alignment, comparison of biological networks is very important in guiding various biological researches. Though we focus on the alignments of two protein-protein interaction networks in this study, BinAligner could be used to align any other types of biological networks, such as gene regulatory networks, metabolic networks and so on. Local network alignment could be used to identify functional components like pathways and complexes that is conserved among different species or individuals, while global network alignment helps to infer the evolutionary relationships among species and could provide some useful information of functional orthologs, which might not be detected from sequence analysis alone. By aligning the PPI networks of KSHV and VZV, we identified a subnetwork consisting of seven orthologous protein pairs and connected by matched links in the two networks. This subnetworks might be conserved for important functions crucial to the two herpesviruses such as virus packing and infection. We also identified some non-orthologous pairs sharing similar link patterns in each network, and might be functional orthologs.

Current version of BinAligner is only feasible for aligning a small network with tens to hundreds of vertices. BinAligner would be useful in accurate comparison of biological networks such as viral networks and in refining sub-network alignment in large network alignments.

However, it is still a big disadvantage for BinAligner to be unscalable. As the sequence similarity comparison and graphlets signature identification are currently available even for networks with thousands vertices and edges [15], the main bottleneck of this method is to generate the exact alignment score of 1-neighborhood networks. There are two main reasons slowing down the process. Firstly, suppose the number of vertices of two networks are *n*_1 _and *n*_2_, then we need to perform *n*_1 _*× n*_2 _pairwise 1-neighborhood subnetwork alignments. The number of comparisons could be huge if both *n*_1 _and *n*_2 _are large. Since each pairwise 1-neighborhood subnetwork comparison is independent with the other, a readily solution is to do parallel programming. Secondly, due to the power-law nature of biological networks, there might be a few vertices with large degrees [19]. However, we only need an estimate of alignment score which could reflect the similarity of 1-neighborhood of two compared core vertices, not the exact alignment. Thus, an heuristic method, such as linear or lagrangian relaxation is a good alternative in this scenario. In the future, parallel programming and heuristic alignments for comparing 1-neighborhoods with the number of vertices in both subnetworks are large will be implemented into BinAligner.

## Conclusion

BinAligner compares the node similarity between biological networks by their sequence similarity, 1-neighborhood subnetwork and similarity on graphlets, and then retrieves a global or local alignment from the node similarity matrix. The results on aligning the PPI networks of two herpes viruses KSHV and VZV show that BinAligner outperforms some existing methods by aligning more orthologous protein pairs and more protein interactions.

### Availability and implementation

BinAligner is available at

http://sysbio.cvm.msstate.edu/BinAligner/.

## Competing interests

The authors declare that they have no competing interests.

## Authors' contributions

XFW and SG supervised the project. JY and JL performed the experiments, analyzed data and wrote the paper. All authors revised the paper.

## Supplementary Material

Additional file 1 **Supplementary file contains the legends and description of 5 supplementary figures and 5 supplementary tables**. Click here for file

## Declarations

This work was partially supported by the Natural Science Foundation of China (No. 10971213) to S.G., and Department of Justice (2010-DD-BX-0596) and National Institutes of Health (NIAID RC1AI086830) to X.-F.W.

This article has been published as part of *BMC Bioinformatics *Volume 14 Supplement 14, 2013: Proceedings of the Tenth Annual MCBIOS Conference. Discovery in a sea of data. The full contents of the supplement are available online at http://www.biomedcentral.com/bmcbioinformatics/supplements/14/S14.
