# Supplementary material for: BinAligner: a heuristic method to align biological networks
Source: BMC Bioinformatics. 2013 Oct 9;14(Suppl 14):S8. doi: 10.1186/1471-2105-14-S14-S8 (PMC3851320; doi:10.1186/1471-2105-14-S14-S8)
Supplement: Additional file 1 — Supplementary file contains the legends and description of 5 supplementary figures and 5 supplementary tables. [file 1471-2105-14-S14-S8-S1.docx]

**Additional file 1**

**Supplementary Online Material**

**BinAligner: a heuristic method to align protein interaction networks**

Jialiang Yang^1,&^, Jun Li^2,&^, Stefan Grünewald^2,*^ and Xiu-Feng Wan^1,*^

^1^Systems Biology Laboratory, Department of Basic Sciences, College of Veterinary Medicine, Mississippi State University, Mississippi State, MS 39762, USA; ^2^CAS-MPG Partner Institute for Computational Biology, Key Laboratory of Computational Biology, Shanghai Institutes for

Biological Sciences, Chinese Academy of Sciences, Shanghai 200031, China

Email:

Jialiang Yang - [jyang@cvm.msstate.edu](mailto:jyang@cvm.msstate.edu);

Jun Li - [lijun@picb.ac.cn](mailto:lijun@picb.ac.cn);

Stefan Grünewald - [stefan@picb.ac.cn](mailto:stefan@picb.ac.cn);

Xiufeng Wan [-wan@cvm.msstate.edu](mailto:-wan@cvm.msstate.edu);

^&^These authors contribute equally to this study

^*^Corresponding author

**Supplementary Figures**

**Figure S1.** The protein-protein interaction network of Kaposi’s sarcoma associated herpes virus (KSHV), in which each node denotes an open reading frame and each link an interaction.

**Figure S2.** The protein-protein interaction network of Varicella zoster virus (VZV), in which each node denotes an open reading frame and each link an interaction.

**Figure S3.** The best alignment graph between KSHV and VZV networks based solely on orthologous information. Each vertex represents a pair of aligned vertices and the orthologous pairs are shaded; a red edge denotes a matched edge, that is, this edge exists in both KSHV and VZV networks.

**Figure S4.** The best pure structure alignment graph of KSHV and VZV PPI networks by BinAligner. An edge in red denotes that it exists in both KSHV and VZV networks.

**Figure S5.** The best alignment graph between KSHV and VZV networks. Each vertex represents a pair of aligned vertices and the orthologous pairs are shaded; there are 3 types of colored edges between two vertices: red, blue and green. A red edge denotes a matched edge, that is, this edge exists in both KSHV and VZV networks. For example, there is a red edge between vertex Orf60/Orf10 and Orf67.5/Orf25, which denotes that Orf60 and Orf67.5 interact in KSHV network and Orf10 and Orf25 interact in VZV network. Similarly, a blue edge denotes the edge existing only in KSHV network while a green edge denotes the edge existing only in VZV network.

**Supplementary Tables**

**Table S1.**  Orthologous pairs between open reading frames of KSHV and VZV.

| **KSHV** | **VZV** | **KSHV** | **VZV** | **KSHV** | **VZV** |
| --- | --- | --- | --- | --- | --- |
| Orf9 | Orf28 | Orf52 | Orf46 | Orf67.5 | Orf49 |
| Orf28 | Orf1 | Orf53 | Orf1 | Orf68 | Orf26 |
| Orf28 | Orf65 | Orf53 | Orf9a | Orf69 | Orf27 |
| Orf29b | Orf42 | Orf53 | Orf65 | Orf72 | Orf7 |
| Orf29b | Orf45 | Orf60 | Orf18 | Orf74 | Orf36 |
| Orf30 | Orf9a | Orf61 | Orf19 | K8 | Orf23 |
| Orf30 | Orf57 | Orf67.5 | Orf25 | K15 | Orf65 |
| Orf39 | Orf50 | Orf67.5 | Orf7 |  |  |
| Orf52 | Orf1 | Orf67.5 | Orf9a |  |  |

*There are overall 57 orthologous pairs in (Kolář *et al*., 2008); 25 of them left after removing the isolated ORFs and the maximum number of non-overlapping orthologous pairs is 16 by solving a simple bipartite matching problem.

**Table S2.**  The best alignment of KSHV and VZV networks based solely on orthologous information

| **KSHV** | **VZV** | **Orth** | **KSHV** | **VZV** | **Orth** | **KSHV** | **VZV** | **Orth** | **KSHV** | **VZV** | **Orth** |
| --- | --- | --- | --- | --- | --- | --- | --- | --- | --- | --- | --- |
| K10 | Orf22 | 0 | Orf23 | Orf25 | 0 | Orf45 | Orf38 | 0 | Orf60 | Orf18 | 1 |
| K10.5 | Orf12 | 0 | Orf25 | Orf44 | 0 | Orf47 | Orf21 | 0 | Orf61 | Orf19 | 1 |
| K11 | Orf43 | 0 | Orf27 | S/L | 0 | Orf48 | Orf66 | 0 | Orf62 | Orf64 | 0 |
| K12 | Orf59 | 0 | Orf28 | Orf1 | 1 | Orf49 | Orf45 | 0 | Orf63 | Orf52 | 0 |
| K15 | Orf65 | 1 | Orf29b | Orf42 | 1 | Orf50 | Orf39 | 0 | Orf65 | Orf61 | 0 |
| K3 | Orf56 | 0 | Orf30 | Orf57 | 1 | Orf52 | Orf46 | 1 | Orf67.5 | Orf49 | 1 |
| K5 | Orf41 | 0 | Orf31 | Orf68 | 0 | Orf53 | Orf9a | 1 | Orf68 | Orf26 | 1 |
| K7 | Orf9 | 0 | Orf34 | Orf17 | 0 | Orf54 | Orf16 | 0 | Orf69 | Orf27 | 1 |
| K8 | Orf23 | 1 | Orf36 | Orf33 | 0 | Orf56 | Orf33.5 | 0 | Orf72 | Orf7 | 1 |
| K8.1 | Orf3 | 0 | Orf37 | Orf53 | 0 | Orf57 | Orf8 | 0 | Orf74 | Orf36 | 1 |
| K9 | Orf24 | 0 | Orf39 | Orf50 | 1 | Orf58 | Orf62 | 0 | Orf75 | Orf32 | 0 |
| Orf2 | Orf34 | 0 | Orf41 | Orf60 | 0 | Orf6 | Orf67 | 0 | Orf9 | Orf28 | 1 |

* 1: orthologous pair; 0: non-orthologous pair.

**Table S3.**  The pure structure alignment of KSHV and VZV networks by BinAligner.

| **KSHV** | **VZV** | **KSHV** | **VZV** | **KSHV** | **VZV** | **KSHV** | **VZV** |
| --- | --- | --- | --- | --- | --- | --- | --- |
| K1 | Orf11 | Orf23 | Orf22 | Orf47 | Orf52 | Orf60 | Orf27 |
| K10.5 | Orf68 | Orf25 | Orf28 | Orf48 | Orf14 | Orf61 | Orf16 |
| K10 | Orf25 | Orf27 | Orf57 | Orf49 | Orf26 | Orf62 | Orf2 |
| K11 | Orf24 | Orf28 | Orf33.5 | Orf50 | Orf23 | Orf63 | Orf61 |
| K12 | Orf1 | Orf29b | Orf60 | Orf52 | Orf38 | Orf65 | Orf55 |
| K15 | Orf36 | Orf30 | Orf12 | Orf53 | Orf4 | Orf67.5 | Orf65 |
| K3 | Orf64 | Orf31 | Orf56 | Orf54 | Orf41 | Orf68 | Orf33 |
| K5 | Orf62 | Orf34 | Orf44 | Orf56 | Orf34 | Orf69 | Orf46 |
| K7 | Orf9 | Orf36 | Orf59 | Orf57 | Orf18 | Orf72 | Orf3 |
| K8.1 | Orf15 | Orf37 | Orf8 | Orf58 | Orf10 | Orf74 | Orf67 |
| K8 | Orf19 | Orf39 | Orf32 | Orf59 | Orf43 | Orf75 | Orf21 |
| K9 | Orf50 | Orf41 | Orf9a | Orf6 | S/L | Orf9 | Orf42 |
| Orf2 | Orf51 | Orf45 | Orf39 |  |  |  |  |

**Table S4.** Comparing the importance of sequence similarity, 1-neighborhood sub-network and graphlets with orthologous information to the network alignment between KSHV and VZV networks.

| $\boldsymbol{\theta}_{\boldsymbol{1}}$ | $\boldsymbol{\theta}_{\boldsymbol{2}}$ | $\boldsymbol{\theta}_{\boldsymbol{3}}$ | **nEdge** | **nOrth** |
| --- | --- | --- | --- | --- |
| 1 | 0 | 0 | 45 | 16 |
| 0 | 1 | 0 | 54 | 9 |
| 0 | 0 | 1 | 53 | 10 |
| 0.9 | 0.1 | 0 | 57 | 16 |
| 0.8 | 0.2 | 0 | 52 | 16 |
| 0.7 | 0.3 | 0 | 51 | 16 |
| 0.6 | 0.4 | 0 | 49 | 16 |
| 0.5 | 0.5 | 0 | 50 | 16 |
| 0.4 | 0.6 | 0 | 50 | 16 |
| 0.3 | 0.7 | 0 | 49 | 16 |
| 0.2 | 0.8 | 0 | 52 | 15 |
| 0.1 | 0.9 | 0 | 56 | 12 |
| 0.9 | 0 | 0.1 | 54 | 16 |
| 0.8 | 0 | 0.2 | 50 | 16 |
| 0.7 | 0 | 0.3 | 49 | 16 |
| 0.6 | 0 | 0.4 | 46 | 16 |
| 0.5 | 0 | 0.5 | 47 | 16 |
| 0.4 | 0 | 0.6 | 45 | 16 |
| 0.3 | 0 | 0.7 | 43 | 16 |
| 0.2 | 0 | 0.8 | 49 | 14 |
| 0.1 | 0 | 0.9 | 50 | 12 |
| 0 | 0.9 | 0.1 | 52 | 10 |
| 0 | 0.8 | 0.2 | 52 | 10 |
| 0 | 0.7 | 0.3 | 57 | 10 |
| 0 | 0.6 | 0.4 | 52 | 10 |
| 0 | 0.5 | 0.5 | 52 | 10 |
| 0 | 0.4 | 0.6 | 52 | 10 |
| 0 | 0.3 | 0.7 | 46 | 10 |
| 0 | 0.2 | 0.8 | 51 | 10 |
| 0 | 0.1 | 0.9 | 54 | 10 |
| 0.9 | 0.09 | 0.01 | 58 | 16 |
| 0.9 | 0.08 | 0.02 | 56 | 16 |
| 0.9 | 0.07 | 0.03 | 56 | 16 |
| 0.9 | 0.06 | 0.04 | 52 | 16 |
| 0.9 | 0.05 | 0.05 | 43 | 16 |
| 0.9 | 0.04 | 0.06 | 54 | 16 |
| 0.9 | 0.03 | 0.07 | 52 | 16 |
| 0.9 | 0.02 | 0.08 | 54 | 16 |
| 0.9 | 0.01 | 0.09 | 51 | 16 |

**Table S5.** The best alignment of KSHV and VZV networks by IsoRank (Singh *et al.,* 2008)

| **KSHV** | **VZV** | **Orth** | **KSHV** | **VZV** | **Orth** | **KSHV** | **VZV** | **Orth** | **KSHV** | **VZV** | **Orth** |
| --- | --- | --- | --- | --- | --- | --- | --- | --- | --- | --- | --- |
| Orf2 | Orf4 | 0 | Orf39 | Orf50 | 1 | Orf59 | Orf24 | 0 | K1 | Orf41 | 0 |
| Orf6 | Orf39 | 0 | Orf41 | Orf21 | 0 | Orf60 | Orf18 | 1 | K3 | Orf22 | 0 |
| Orf9 | Orf28 | 1 | Orf45 | Orf53 | 0 | Orf61 | Orf19 | 1 | K5 | Orf62 | 0 |
| Orf23 | Orf56 | 0 | Orf47 | Orf61 | 0 | Orf62 | Orf32 | 0 | K7 | S/L | 0 |
| Orf25 | Orf15 | 0 | Orf48 | Orf11 | 0 | Orf63 | Orf33 | 0 | K8 | Orf23 | 1 |
| Orf27 | Orf17 | 0 | Orf49 | Orf45 | 0 | Orf65 | Orf52 | 0 | K8.1 | Orf67 | 0 |
| Orf28 | Orf65 | 1 | Orf50 | Orf34 | 0 | Orf67.5 | Orf25 | 1 | K9 | Orf1 | 0 |
| Orf29b | Orf42 | 1 | Orf52 | Orf46 | 1 | Orf68 | Orf26 | 1 | K10 | Orf60 | 0 |
| Orf30 | Orf57 | 1 | Orf53 | Orf9a | 1 | Orf69 | Orf27 | 1 | K10.5 | Orf44 | 0 |
| Orf31 | Orf10 | 0 | Orf54 | Orf12 | 0 | Orf72 | Orf7 | 1 | K11 | Orf16 | 0 |
| Orf34 | Orf3 | 0 | Orf56 | Orf59 | 0 | Orf74 | Orf36 | 1 | K12 | Orf33.5 | 0 |
| Orf36 | Orf43 | 0 | Orf57 | Orf38 | 0 | Orf75 | Orf9 | 0 | K15 | Orf64 | 0 |
| Orf37 | Orf68 | 0 | Orf58 | Orf8 | 0 |  |  |  |  |  |  |

**References**

S1 Kolář, M., Lässig M. and Berg J. (2008) From protein interactions to functional annotation: graph alignment in *Herpes. BMC Systems Biology, 2:90*

S2 Singh, R., Xu, J. and Berger, B. (2008) Global alignment of multiple protein interaction networks with application to functional

orthology detection, *PNAS*, **105**: 12763-12768.
